# Focus on the Role of D-serine and D-amino Acid Oxidase in Amyotrophic Lateral Sclerosis/Motor Neuron Disease (ALS)

**DOI:** 10.3389/fmolb.2018.00008

**Published:** 2018-02-13

**Authors:** Nazanin R. Kondori, Praveen Paul, Jacqueline P. Robbins, Ke Liu, John C. W. Hildyard, Dominic J. Wells, Jacqueline S. de Belleroche

**Affiliations:** ^1^Neurogenetics Group, Division of Brain Sciences, Department of Medicine, Imperial College London, London, United Kingdom; ^2^Neuromuscular Diseases Group, Department of Comparative Biomedical Sciences, Royal Veterinary College, London, United Kingdom

**Keywords:** Amyotrophic lateral sclerosis, motor neurons, D-amino acid oxidase, survival study, motor phenotype, motor neuron degeneration, excitotoxicity, NMDA receptors

## Abstract

We have investigated a pathogenic mutation in D-amino acid oxidase (DAO), DAO^R199W^, associated with familial Amyotrophic Lateral Sclerosis (ALS) that impairs D-serine metabolism and causes protein aggregation, autophagy and cell death in motor neuron cell lines. These features are consistent with the pathogenic processes occurring in ALS but most importantly, we have demonstrated that activation of the formation of ubiquitinated protein inclusions, increased autophagosome production and apoptotic cell death caused by the mutation in cell lines are attenuated by 5,7-dichlorokynurenic acid (DCKA), a selective inhibitor of the glycine/D-serine binding site of the NMDA receptor. D-serine is an essential co-agonist at this glutamate receptor. This data provides insight into potential upstream mechanisms that involve the action of D-serine at the NMDA receptor and might contribute to neurodegeneration. This is highly relevant to sporadic ALS (SALS), familial ALS, as well as ALS models, where elevated levels of D-serine have been reported and hence has broader clinical therapeutic implications. In order to investigate this further, we have generated a transgenic line expressing the pathogenic mutation, in order to determine whether mice expressing DAO^R199W^ develop a motor phenotype and whether crossing the SOD1^G93A^ model of ALS with mice expressing DAO^R199W^ affects disease progression. We found that heterozygous expression of DAO^R199W^ led to a significant loss of spinal cord motor neurons at 14 months, which is similar to that found in homozygous mice expressing DAO^G181R^. We hypothesize that DAO has potential for development as a therapeutic agent in ALS.

## Introduction

The focus of this mini-review is to make the case for the potential involvement of D-serine in neurodegenerative diseases which involves impaired activity of D-amino acid oxidase (DAO), the enzyme responsible for its degradation, and is most strongly indicated in Amyotrophic lateral Sclerosis ALS (ALS). On the other hand, we propose that in brain regions such as cerebral cortex, where levels of DAO are very limited, another mechanism may be involved which is mediated through the up-regulation of serine racemase (SR), the biosynthetic enzyme responsible for D-serine biosynthesis. Both mechanisms result in an upregulation of D-serine caused either by impaired metabolism of D-serine by DAO or increased biosynthesis by SR.

### Background

Amyotrophic Lateral Sclerosis (ALS), the most common adult-onset neuromuscular disease, is a highly debilitating, rapidly progressing condition that causes muscle atrophy, paralysis, impaired speech and swallowing, with death usually occurring within 3–5 years of diagnosis. These clinical features reflect the fundamental changes that occur in the motor neuron populations of the spinal cord, brain stem and motor cortex that mediate these essential motor functions. The typical neuropathological features of ALS are characterized by the accumulation of cytosolic ubiquitinated protein inclusions, most of which are positive for TDP-43, encoded by *TAR DNA binding protein 43* (*TARDBP)* (Neumann et al., 2006). To-date, no effective treatments have been discovered and new therapeutic approaches are urgently needed.

Identification of mutations in the familial form of the disease (FALS), which accounts for about 10% of ALS cases, has led the way in the elucidation of disease mechanisms in both the sporadic form and familial forms of the disease. To date, mutations have been identified in approximately 70% of FALS kindred, with the most common FALS genes being *C9orf72, Superoxide dismutase-1 (SOD1), TARDBP* and *Fused in Sarcoma (FUS)*. Most importantly, it has emerged that the majority of pathogenic mutations in FALS occur in genes encoding proteins involved in RNA processing and proteostasis, covering protein folding, the unfolded protein response involved in removal of damaged/mutant proteins, protein trafficking and protein degradation through proteasomal and autophagic pathways, all of which play a fundamental role in normal motor neuron function (Chen and de Belleroche, 2012). Studies on the effects of these mutations associated with FALS has provided enormous insight into the molecular mechanisms of disease which provides a valuable framework of targets for potential drug development (Taylor et al., 2016).

We recently reported a coding mutation in *DAO* (DAO^R199W^) associated with FALS, lacking all previously identified mutations (Mitchell et al., 2010). Interestingly, this mutation provides a novel input into disease mechanisms, as it provides an “upstream” trigger that may potentiate cell death in vulnerable motor neurons. This will be the main focus of this mini review.

It has only relatively recently been established that D-amino acids such as D-serine, D-alanine, and D-proline are found in man, present in multiple tissues including the brain and spinal cord. Of particular importance, D-serine is known to play a crucial role in synaptic plasticity, where D-serine is thought to be the major co-agonist at the NMDA receptor mediating Long Term Potentiation (LTP) (Mothet et al., 2000, 2005; Yang et al., 2003; Panatier et al., 2006; Li et al., 2013). Detailed analysis of the crystal structure of human DAO has highlighted the structural basis for marked differences seen in the properties of the human protein compared to porcine DAO, with which it shares 85% sequence identity, e.g., weaker binding to FAD and a slower rate of Flavin reduction, and has established that these effects are due to a hydrophobic stretch of amino acids (VAAGL) that lie at the si-face that exists in a different conformation (Kawazoe et al., 2006).

**Figure 1:**
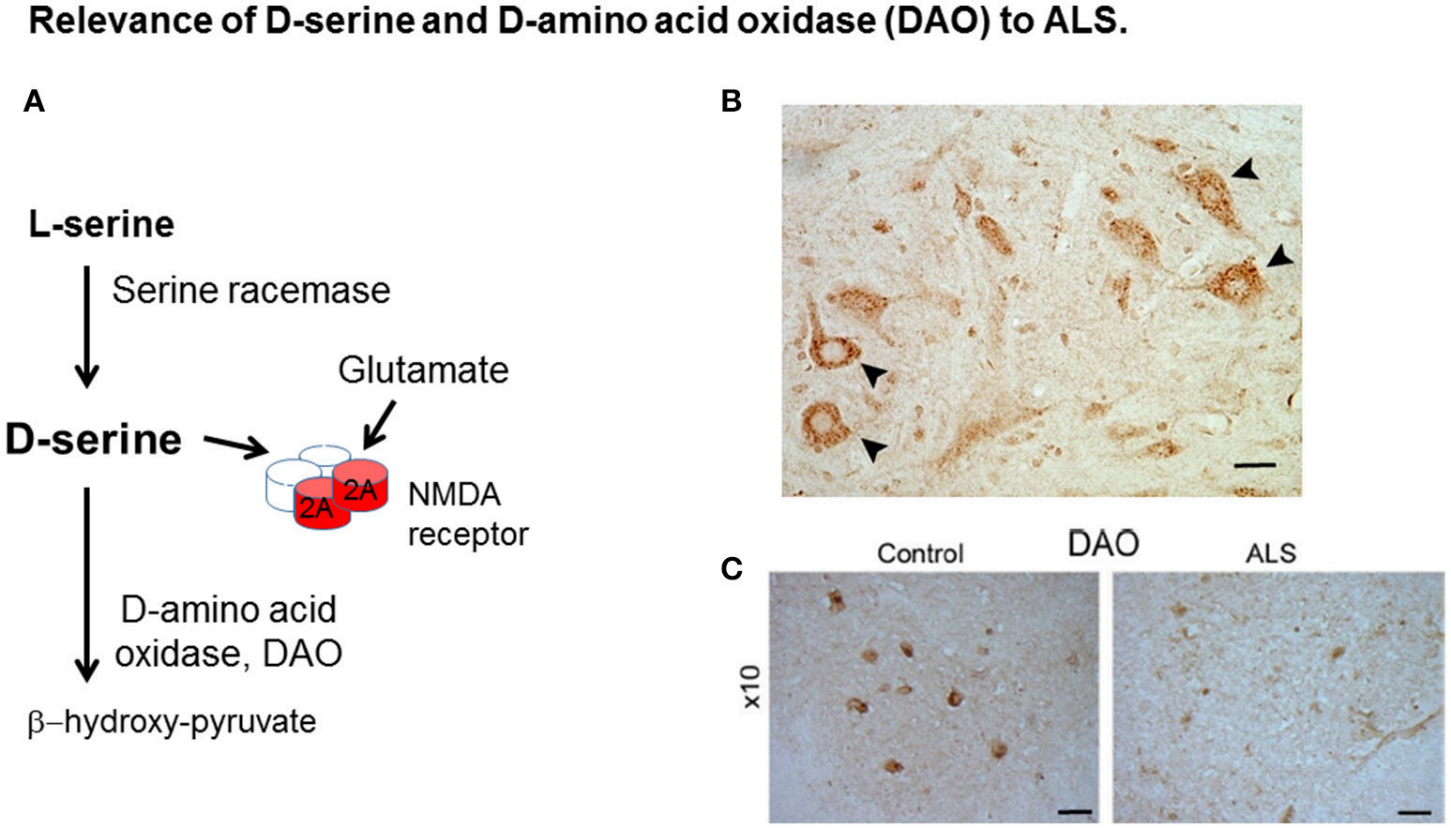
Relevance of D-serine and D-amino acid oxidase (DAO) to ALS. **(A)** D-serine is synthesized from L-serine by serine racemase and is metabolized by DAO. D-serine is a co-agonist with glutamate at the NMDA receptor (NMDAR). **(B)** D-serine in spinal cord motor neurons. Localisation of D-serine immunoreactivity in mouse lumbar spinal cord. D-serine immunoreactivity is widespread in gray matter including motor neurons (arrowheads). Scale bar is 50 μm. Data is taken from Paul and de Belleroche (2012) with permission from Springer. **(C)** DAO-IR in human spinal cord. DAO is highly concentrated in motor neurons in spinal cord and brain stem which are depleted in ALS. Distribution of DAO in lumbar spinal cord. Lumbar sections (L5) from control and amyotrophic lateral sclerosis (ALS) cases were stained for DAO. Scale bar is 100 μm. Data taken from Paul et al. (2014) with permission from Elsevier.

It has been established that D-serine is derived from L-serine through the enzyme serine racemase (SR) (Wolosker et al., 1999) (Figure 1A). This reaction is known to occur mainly in neuronal cells, while L-serine is synthesized in astrocytes from 3-phosphoglycerate and is actively shuttled to neurons through the transporters, such as ASCT1. Mammalian SR has a type II beta eliminase fold and the enzyme catalyses both the beta elimination of L-serine and its racemization to D-serine, the mechanism of which has been further elucidated in recent human SR structure/activity relationship studies (Nelson et al., 2017).

Levels of synaptic D-serine and L-serine are regulated predominantly by three transporters, the Na^+^-independent alanine-serine-cysteine 1 transporter (Asc-1) which is a high affinity neuronal transporter selective for D- and L- amino acids, the Na^+^-dependent alanine-serine-cysteine-threonine 2 transporter (ASCT2) with higher affinity for L-amino acids than D-amino acids and alanine-serine-cysteine-threonine 1 transporter (ASCT1) which has a high affinity for small neutral L amino acids. Studies on the effects of the ^G93A^SOD1 mutation on the activity of these transporters in a motor neuron cell line, NSC-34, showed that D-serine uptake was significantly impaired by the mutation, where the K_M_ for D-serine was increased but no change occurred in V_max_ (Lee et al., 2017). In contrast, L-serine uptake was increased. Both effects were Na^+^ dependent and small interfering RNA studies showed that both ASCT2 (SLC1A5) and ASCT1 (SLC1A4) mediated these effects.

### Why is DAO relevant to a neurodegenerative condition?

The finding of a mutation in DAO, which is transmitted with disease offered the opportunity to elucidate pathogenic disease mechanisms caused by the mutation. Indeed, both D-serine and DAO, are strongly expressed in spinal cord and brain stem (Horiike et al., 1994; Mitchell et al., 2010; Paul et al., 2014), regions involved in ALS pathogenesis (Figures 1B,C, respectively). Furthermore DAO enzyme activity is substantially impaired by the presence of the mutation in spinal cord from a case expressing this mutation compared to control tissue and also in cell lines expressing DAO^R199W^ compared to those expressing DAO^WT^ (Mitchell et al., 2010; Paul and de Belleroche, 2012; Paul et al., 2014). Most importantly, D-serine has been shown to be elevated in spinal cord, not only in sporadic cases of ALS but also in the SOD1^G93A^ mouse model of ALS (Sasabe et al., 2007).

**Figure 2:**
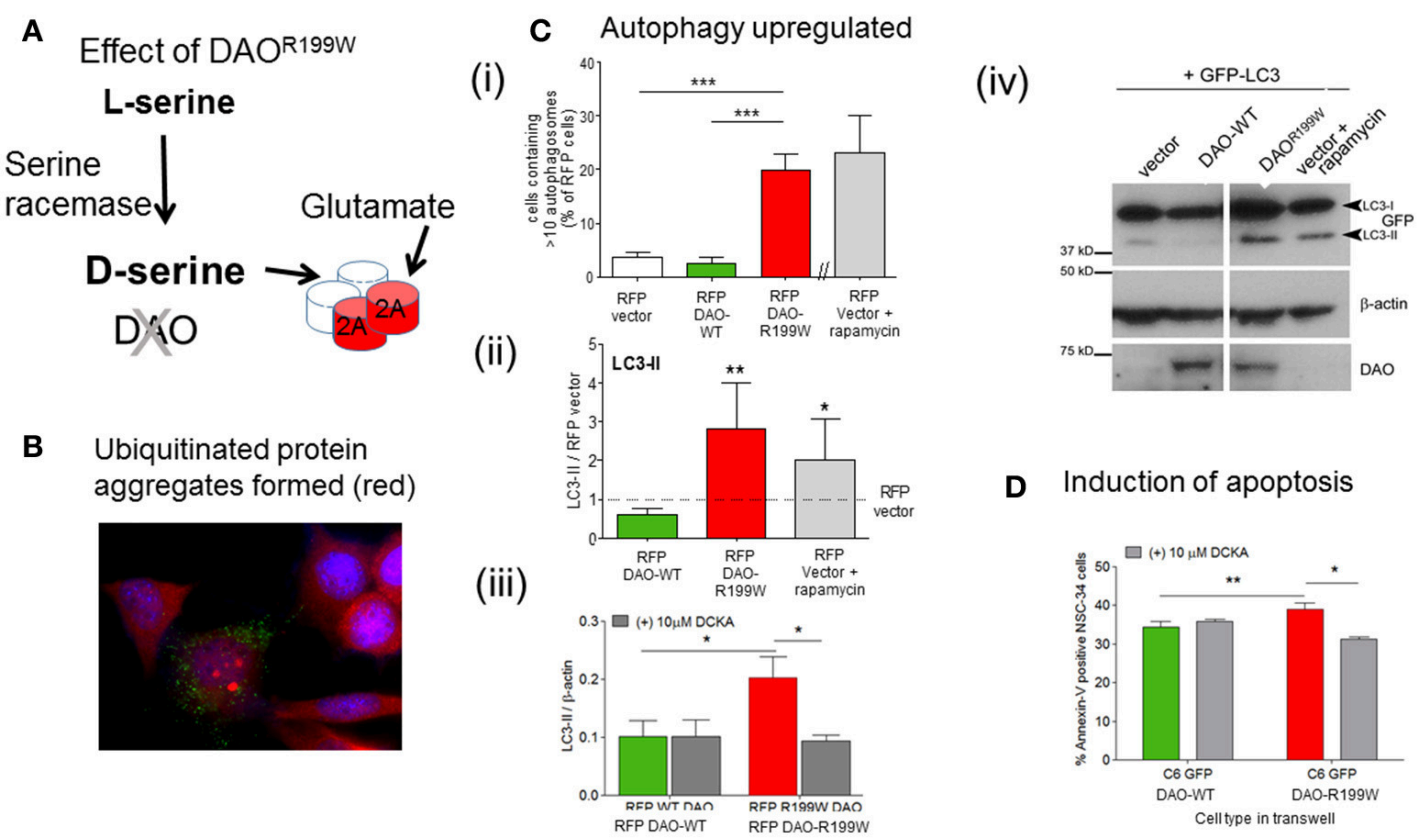
Pathogenic processes caused by FALS-associated mutation DAO^R199W^. **(A)** Model showing the potential effects of DAO^R199W^. **(B)** Effect of DAO^R199W^ on ubiquitin aggregates. NSC-34 cells expressing GFP-tagged DAO 72 h after transfection. Ubiquitin (UBQ) staining (red) with aggregates in GFP-positive cells are shown in a merged image with DAPI nuclear staining. Data taken from Mitchell et al. (2010). **(C)** DAO^R199W^ promotes autophagy. **(i)** NSC-34 cells were co-transfected with RFP-tagged D-amino acid oxidase (DAO) and GFP-tagged protein light chain 3 (LC3). The number of vector or DAO transfected cells containing more than 10 GFP-LC3 puncta or autophagosomes were quantified. **(ii)** Quantification of LC3 (I and II) using Western blot analysis. Rapamycin induced autophagy was used as a positive control. Levels of LC3-II protein were calculated using densitometry and normalized to protein levels of RFP-vector. **(iii)** NSC-34 cells were treated with 5,7-dichloro-4-hydroxyquinoline-2-carboxylic acid (DCKA), immunoblotted and quantified. Significant 1-way ANOVA with Friedman's test subject to post hoc testing with Dunn's multiple comparison test or 2-way analysis of variance (ANOVA). Values are means ±SEM for 4–6 experiments, for p values, ^*^p < 0.05; ^**^p < 0.01; ^***^p < 0.001. The images shows RFP-DAO (red), ubiquitin (green), and DAPI nuclear stain (blue). DAPI, 4′,6-diamidino-2-phenylindole. Data taken from Paul et al. (2014) with permission from Elsevier. **(iv)** A representative western blot is shown. **(D)** DAO^R199W^ promotes apoptosis in neuronal cells. Annexin V levels in NSC-34 neuronal cells co-cultured with C6 glial cells permanently expressing wild-type (WT) or DAO^R199W^, treated with vector or 5,7-dichloro-4-hydroxyquinoline-2-carboxylic acid (DCKA). Paired *t*-test used. Values are means ± standard error of the mean, for 3 experiments with *p*-values shown, ^*^p < 0.05, ^**^p < 0.01. Data taken from Paul et al. (2014) with permission from Elsevier.

The functional effects of the presence of DAO^R199W^ were extensively investigated using a motor neuron cell line, NSC-34, in order to determine whether the mutation influenced proteostasis and cell viability (Figure 2A). Furthermore, expression of DAO^R199W^ caused increased ubiquitinated protein aggregate formation (Figure 2B), which was associated with significantly increased formation of autophagosomes compared to DAO^WT^ (Figure 2Ci), and increased levels of LC3-II, a key marker of increased autophagy (Figure 2Cii). This effect was significantly attenuated by the selective D-serine/glycine site antagonist, 5,7-dichloro-4-hydroxyquinoline-2-carboxylic acid (DCKA) (Figure 2Ciii), determined by Western blot analysis (Figure 2Civ). Effects on cell viability were analyzed by measurement of Annexin V, using Flow cytometry, and showed a significant activation of apoptosis in the mutant cell line compared to controls (Figure 2D; Paul et al., 2014). These results demonstrated significant effects of the mutation to promote aggregate formation, with increased autophagy and apoptosis, all of which are consistent with neuropathological features found in ALS cases.

Structure-function relationships have been studied in human DAO mutations associated with ALS and other coding substitutions by Cappelletti et al. (2015) and have provided valuable information about the effects of these mutations. The DAO^R199W^ substitution altered protein conformation and enzyme activity was lost, which resulted in an abnormal increase in cellular D-serine levels, which could contribute to the protein instability and decrease in kinetic efficiency seen in ALS and potentially lead to increased synaptic D-serine. Reduced enzyme activity has also been reported in spinal cord from an ALS case harboring this mutation (Mitchell et al., 2010), Additional studies on the human DAO^G183R^ mutation that corresponds to the G181R substitution found in a strain of ddY mice lacking DAO activity also alters protein conformation and negatively affects the ability of the apoprotein to bind to the flavin cofactor in the orientation required for hydride-transfer during catalysis. At the cellular level, the overexpressed DAO^G183R^ was not fully targeted to peroxisomes, formed protein aggregates colocalised with ubiquitin, and significantly increased both the cellular concentration of D-serine and the D/(D+L)-serine ratio. As pointed out by Murtas et al. (2017), while both the DAO^R199W^ mutation associated with ALS and the DAO^G331V^ variant related to schizophrenia susceptibility led to the formation of protein aggregates and apoptosis, DAO^G183R^ over-expression did not induce apoptosis.

The next conundrum to solve was to determine whether the impaired level of DAO activity contributed to the pathogenic process through reduced metabolism of D-serine and hence build-up of D-serine, which could regulate the activity of glutamate at the NMDA receptor and hence potentiate excitotoxicity and cell death. Alternatively, the presence of the mutant protein alone could be responsible for activating apoptosis, without requiring the action of extracellular D-serine at the NMDA receptor. In order to explore this mechanism, we used a co-culture technique in which cells expressing DAO^R199W^ were placed in a trans-well above NSC-34 cells separated by a membrane (0.4 mm filter), in order to determine whether an extracellular factor released from cells expressing DAO^R199W^ and not DAO^WT^ or control cells would influence survival. Interestingly, we found that apoptosis was sensitive to attenuation by 5,7-dichlorokynurenic acid (DCKA), a selective inhibitor of the glycine/D-serine binding site of the NMDA receptor (Figure 2Ciii; Paul et al., 2014). Thus, this data provided insight into upstream mechanisms that involve the release of D-serine from neuronal and glial cells and lead to neurodegeneration, which is mediated by the NMDA receptor.

### Is motor neuron function affected by impaired DAO activity?

Characterisation of the effects of the *in vivo* expression of an ALS-linked mutation in DAO on phenotype and loss of spinal cord motor neurons was carried out using DAO^R199W^ transgenic mouse lines overexpressing DAO^R199W^, created using human DAO^R199W^, driven by a chicken β-actin promoter and cytomegalovirus enhancer. The DAO^R199W^ transgene was present in spinal cord and cerebral cortex at concentrations that were 40-fold greater than those of mouse DAO, determined by qPCR using human and mouse specific primers (Kondori et al., 2017). DAO protein was found to be widely expressed in heart, muscle, brain and spinal cord. Immuno-histochemical staining of DAO in DAO^R199W^ transgenic mice showed evidence of increased ubiquitin staining compared to wild-type mice (Kondori et al., 2017).

The development of ALS related disabilities were assessed in both hind legs twice weekly for each mouse from approximately 30 days of age in DAO^R199W^ transgenic mice. Neurological scores were assigned using a scale of 0–4, established by the ALSTDI through detailed observations of SOD1^G93A^ mouse pathology (Scott et al., 2008; Gill et al., 2009; Lincecum et al., 2010), where 0 was the full extension of hind legs and 4 indicated the inability to right itself within 20 s. Body weight, motor features and gait were monitored in a large cohort for one year blind to genotype (Kondori et al., 2017) as previously described (Vincelette et al., 2007; Sharp et al., 2008; Kaneb et al., 2011).

A distinct feature seen in DAO^R199W^ mice, which are heterozygous for the mutation, was kyphosis, a dorsal curvature of the spine due to progressive rigid thoracolumbar kyphosis, caused by a loss of muscle tone, secondary to motor neuron degeneration. This was most conspicuous in females. Kyphosis is also a feature of *SOD1*^*G*93*A*^ (Azzouz et al., 1997), *SOD1*^*G*37*R*^ (Filali et al., 2011), and *Tardbp* targeted inactivation ALS mouse models (Wu et al., 2012). Animals expressing the *DAO*^*R*199*W*^ transgene consistently had a lower body weight at each age compared to wild type littermates (*n* = 15–23 per gender per genotype). Kyphosis was not detected in any wild-type animals.

In order to determine whether the presence of the *DAO*^*R*199*W*^ mutation affected motor neuron populations, ventral horn motor neurons in the lumber region (L3-L5) of the spinal cord of DAO^R199W^ and control littermates were quantified at 14 months (*n* ≥ 5 per genotype, per sex) and showed a significant reduction of 19% (*p* < 0.023) in motor neuron numbers in mice carrying the mutant allele in comparison to their wild type littermates. When separated according to gender, a significant decrease (25.7%) was found in females (*p* = 0.0132) but in males a smaller non-significant reduction was obtained (16.5%).

It was also interesting to compare these findings with those obtained by Sasabe et al. (2012) using a mouse line that was homozygous for a naturally occurring mutation, DAO^G181R^ (Konno and Yasumura, 1983), that severely impairs DAO enzyme activity. To obtain a homozygous line, ddY/DAO^−^ was initially backcrossed with C57BL/6J mice. In these animals, abnormal reflexes characterized by retraction of hind limbs were evident, as reported in the SOD1^G93A^ mouse model of ALS, together with a reduction in motor neuron number of 24% in DAO^−/−^ in mice at 8 months. By 15 months, axonal degeneration with muscle atrophy was detected together with increased ubiquitination in spinal cord. Suppression of DAO activity was also observed in the reticulospinal tract, a pathway that plays an important role in regulating motor neuron excitability in animal models of ALS (Sasabe et al., 2012).

In summary, while a mutation can cause a lethal disease in humans, no overt ALS phenotype was evident in mice expressing DAO^R199W^. However, marked structural and abnormal motor features were evident which were associated with a significant loss of lumbar motor neurons.

Triggering a “human” phenotype in mouse models is not always possible (e.g., absence of upper motor neuron signs in ALS models, lack of neurodegeneration in a VAPB transgenic model of ALS; Tudor et al., 2010). Hence we used another approach and investigated whether the *DAO*^*R*199*W*^ mutation would potentiate the effects of the SOD1 mouse model of ALS.

### Does the presence of DAO^R199W^ potentiate the effect of the SOD1^G93A^ mutation on motor function and survival?

To investigate the effect of overexpression of DAO^R199W^ on the development of the ALS-like phenotype and survival in the SOD1^G93A^ mouse model of ALS, these two lines were crossed to generate four genotypes, controls lacking both transgenes, SOD1^G93A^ mice carrying a single transgene, DAO^R199W^ mice expressing a single transgene and double transgenic animals (DAO^R199W^/SOD1^G93A^). A range of tests were performed on each cohort, including weight measurement, onset of neurological deficits, gait analysis and survival. The cohort size for each cohort was >14 males and >14 females. Neurological scores for both hind legs were assessed twice a week for each mouse in a “blinded fashion” (scale of 0–4), developed by ALSTDI. A significantly earlier onset of neurological signs was seen in female SOD1^G93A^/DAO^R199W^ females compared to SOD1^G93A^ females (*p* = 0.0239; Kondori et al., 2017). As previously shown, significantly earlier onset of neurological signs was found in SOD1^G93A^ males compared to SOD1^G93A^ females (*p* = 0.0004).

In conclusion, these results suggest that impaired DAO activity may increase D-serine levels and potentiate possible excitotoxic effects mediated through the NMDA receptor. In contrast enhanced levels of DAO may have beneficial effects. This approach clearly has enormous potential for therapeutic application to man in the treatment of both sporadic cases and familial cases of ALS. Indeed the first drug licensed for treatment of ALS, riluzole, which increases survival by 3–4 months but does not confer long-term protection (Traynor et al., 2003) is known to target glutamate release. However, a drug targeting DAO would introduce an important degree of specificity in that its effects would be focused on the D-serine/glycine binding site of the NMDA receptor. Indeed, AAV administration of DAO in SOD1^G93A^ mice has now been shown to reduce motor neuron loss. Overexpression of DAO in SOD1^G93A^ mice was carried out using a single injection of single stranded adeno-associated virus serotype 9 (ssAAV9) vector (1 μl/g body weight (titre of 10^12^/ml) intrathecally into lumbar region at 90 days (Wang et al., 2017), which alleviated motor neuron loss and glial activation and extended survival. Although only moderate expression of DAO was obtained, 2-fold compared to GFP vector injected and non-transgenic controls, a significant beneficial effect could be obtained in the combined cohort of males and females. When split by gender this effect was only significant in females (*p* = 0.004).

### Relevance of DAO to ALS pathogenesis

This was also borne out in a recent comprehensive exome sequencing study in 2,874 SALS cases and 6,405 controls, using collapsing analysis where the gene acts as the unit of analysis. This study identified TANK-binding kinase 1 (*TBK1*) as a new risk gene for ALS. *TBK1* is a kinase that phosphorylates Sequestosome 1 *(SQSTM1*) and Optineurin (*OPTN*), two components of autophagy enabling autophagosome generation. Interestingly, in addition, the authors showed that DAO was the only known predisposition gene where the presence of DNA variants was significantly associated with clinical outcome, decreasing rates of survival (Cirulli et al., 2015).

A further link between key players in ALS pathogenesis, namely, the RNA binding proteins (RBPs) and DAO has also emerged recently. RBPs (TARDBP, FUS, TAF15) play an important role in RNA processing and are strongly implicated in ALS, as mutations in these genes cause FALS. Recently, a mutation in the RBP hnRNP A2/B2 (D290V) was identified that, in common with mutations in the FALS gene VCP, causes multisystem proteinopathy. hnRNP A2/B2 targets UAGG motifs in 564 genes affecting RNA processing, splicing and polyadenyation (targeting 3′UTR rather than introns). Both the pathogenic mutation and knockdown of this gene led to abnormal splicing changes (Martinez et al., 2016). Using splicing microarray analysis, the most significant and robust alternative splicing event seen after depletion of hnRNP A2/B1 was exon 9 skipping in DAO (a 118 nucleotide cassette). This short form of DAO (DAO-short) lacks 2 alpha helices and 3 beta sheets due to a reading frameshift and early truncation of the protein, severely reducing enzyme activity. The effect of hnRNP A2B1 knockdown (ASO) on expression of the alternative splice (AS) variant (short) was to cause an increase in AS mRNA but a decrease in AS protein. Furthermore, stably expressing cell lines for each isoform showed reduced protein levels in DAO-short despite similar transcript levels of each isoform (Martinez et al., 2016).

In summary, depletion of hnRNP A2/B1 protein substantially lowered the abundance of the longer (constitutive) transcript compared to the shorter spliced isoform. In contrast to the constitutive form, the shorter isoform of DAO exhibited reduced enzymatic activity and reduced D-serine metabolism. Thus, these effects of hnRNP A2/B1 knockdown on the regulation of splicing of DAO are consistent with a potential DAO-mediated mechanism of pathogenicity and the lower levels of hnRNP A2/B1 observed in ALS. Overall these findings are of considerable importance as they highlight a potent regulatory mechanism that could underpin the neurodegenerative process through the switch between transcripts yielding stable proteins to those yielding unstable proteins subject to rapid degradation.

## Conclusion

From the data presented in this mini-review, it can be seen from a wide range of experimental approaches that evidence is accumulating for an important role of DAO in ALS. Studies carried out both in cell culture and *in vivo* show that expression of DAO carrying a mutation associated with FALS causes a range of pathological features characteristic of ALS. Furthermore, genetic analysis implicates a significant association between the abundance of DAO variants and ALS severity. In addition, the regulatory effects of the RNP, hnRNP A2/B1, reported on DAO splicing, suggest a possible mechanism of ALS pathogenesis.

## Ethics statement

All animal experiments were carried out under license from the Home Office (UK) in accordance with the Animals Scientific Procedures Act 1986 and were approved by Imperial College London / Royal Veterinary College ethical review committees.

## Author contributions

All authors listed have made a substantial, direct and intellectual contribution to the work, and approved it for publication.

### Conflict of interest statement

The authors declare that the research was conducted in the absence of any commercial or financial relationships that could be construed as a potential conflict of interest.
